# Radiation dosimetry effect evaluation of a carbon fiber couch on novel uRT-linac 506c accelerator

**DOI:** 10.1038/s41598-021-92836-2

**Published:** 2021-06-29

**Authors:** Dazhen Jiang, Zhen Cao, Yongchang Wei, Tingting Cao, Jiuling Shen, Conghua Xie, Yunfeng Zhou, Hui Liu, Jun Zhang

**Affiliations:** 1 Department of Radiation and Medical Oncology, Hubei Key Laboratory of Tumor Biological Behaviors, Hubei Cancer Clinical Study Center, Zhongnan Hospital of Wuhan University, Wuhan, 430071 Hubei People’s Republic of China; 2 School of Physics and Technology, Wuhan University, Wuhan, 430072 People’s Republic of China

**Keywords:** Radiotherapy, Radiotherapy, Techniques and instrumentation

## Abstract

Recently, a diagnostic helical CT is integrated into a linear accelerator, called uRT-linac 506c, whose CT scanning dataset can be directly used to do simulation. This novel structure provides a possibility for online adaptive radiotherapy. For adaptive radiotherapy, the carbon fiber couch is an essential external device for supporting and positioning patients. And the effect on dose attenuation and distribution caused by a couch is inevitable and vital for precise treatment. In this research, the couch equipped with uRT-linac 506c was evaluated on the radiation dosimetry effect. The treatment couch equipped on the uRT-linac 506c accelerator was evaluated, and its effect on the attenuation, surface dose and dose buildup were measured for different phantom positions (offset = 0 cm, offset =  + 10 cm and offset =  − 10 cm, respectively) and different gantry angles. Since uRT-linac 506c is exclusively capable to provide diagnostic CT scanning data with real relative electron density (RED), this CT scanning data of the couch can be used directly in uRT-TPS to design plans. This scanned couch dataset was designated as the model A. The model B was a dummy structure of a treatment couch inserted with artificially preset RED. The dose calculation accuracy of these two models was compared using PB, CC, and MC on uRT-TPS. With the effect of carbon fiber couch, the surface dose was increased at least 97.94% for 25 × 25 cm^2^ field and 188.83% for 10 × 10 cm^2^ field, compared with those without. At different phantom positions (offset = 0, + 10, − 10 cm), the attenuation for 6 MV photon beam at gantry angle 180° were 4.4%, 4.4%, and 4.3%, respectively, and varied with changes of gantry angle. There do exists dose deviation between measurement and TPS calculation with the involvement of treatment couch, among the three algorithms, MC presented the least deviation, and the model A made less and steadier deviation than the model B, showing promising superiority. The attenuation, surface dose, and buildup effects of the carbon fiber couch in this study were measured similarly to most counterparts. The dose deviation calculated based on the couch dataset scanned by the diagnostic helical CT was smaller than those based on a dummy couch. This result suggests that an accelerator equipped with a diagnostic CT, which can help reduce the dose deviation of the carbon fiber couch, is a promising platform for online adaptive radiotherapy.

## Introduction

External beam radiotherapy (EBRT) requires a treatment couch equipped on medical linear accelerator to support patient and facilitate positioning, which is supposed to be sturdy, rigid, light weight and most importantly radio-transparent. Carbon fiber material fits all the criteria and is currently made for treatment couch on almost every prevalent linear accelerator^1, 2^. Compared with other types of treatment couch such as the tennis-racket type, that of carbon fiber is capable to avoid radiation dose deviation in radiotherapy produced by bed sagging influence^3^. A carbon fiber couch is typical of a sandwich design, for which two layers of 2–4 mm in thickness make up the top and bottom of the couch, the middle layer is of near air equivalent material^4^. Even though carbon fiber has been assumed to be radiotranslucent, intensive studies still report non-negligible attenuation or build-up affection of radiation dose caused by it^5–8^. With the goal to deliver prescribed dose to targeted tumor volume with high conformal distribution and sparing the adjacent organs at risk (OAR), modern techniques of radiation therapy, like intensity modulated radiotherapy (IMRT), imaged guided radiotherapy (IGRT) and volumetric arc therapy (VMAT), utilize rotation of the gantry and perform the irradiation around the target from different directions^9–11^. Therefore, the effect of treatment couch on dose distribution becomes complicated and worth elaborative research on it, especially for every specific one before being put into service^6, 12^.

The AAPM TG176 summarized a review of many studies on the dosimetry effect about external devices, including the couch^8^. They concluded that the range of attenuation through the carbon fiber couch was between 2 and 6%. Njeh et al.^13^ determinated the photon beam attenuation of Brainlab imaging couch, and verified the various angles and field sizes had effect on attenuation by 4.9% and 3.4% for 5 × 5 cm^2^ and 10 × 10 cm^2^ field sizes respectively with 6 MV photons. Gerig et al.^14^ had demonstrated that different treatment couch causes non-negligible beam attenuation ranging from 4 to 9%. Butso et al.^15^ reported the variations at skin dose with linac bed material, and showed that basal cell layer dose was increased up to 51% of maximum dose with a carbon fiber couch and 28% for a tennis string/Mylar couch when compared to anterior beams. Many other literatures^16–18^ has verified that the attenuation of couch had an effect of the surface dose and the depth-dose curves with different energy beam. Pulliam et al.^12^ demonstrated the dose loss over 3% to both the clinical and planning target volumes, and also reduce the coverage of PTV when couch attenuation was excluded from the dose calculation. Therefore, the effect of carbon fiber treatment couch on dose distribution must not be ignored, and it is vital to explore it thoroughly in clinical practice^12, 19^.

The uRT-linac 506c medical linear accelerator (United imaging HealthCare co., LTD.) is an innovative type of accelerator, which combines a diagnostic helical CT with high dose rate intensity modulated accelerator to make it capable to perform precise radiotherapy with high resolution CT image verification. This novel structure provides a possibility for online adaptive radiotherapy, 4D image guided radiotherapy (IGRT), and so on. Since the diagnostic CT machine is sequentially located behind the accelerator, the couch stepping depth is remarkably longer than that of other counterparts, which demands more rigid, sturdier and steadier couch than ever. It could make the treatment couch on the uRT-linac 506c behave very differently from a common one for the dose distribution during modern precise radiotherapy. In this work, we are aiming to evaluate the detailed radiodosimetry effect of the uRT-linac 506c carbon fiber couch on dose attenuation, surface dose and buildup, and TPS calculation as well, to provide helpful information or guidance for those who might use this accelerator in the future.

There are generally two different approaches employed to include the couch in the treatment planning process presently^8^. The first one integrates a single separate CT scan of the treatment couch into the patients’ CT dataset by TPS fusion modules. The defect of this method is the bias caused by the fusion modules of the couch CT and the planning dataset. However, the uRT-linac 506c accelerator, whose scanned CT dataset can be used for simulation, can solve this problem by directly designing plan based on the dataset containing a couch scanned by the diagnostic helical CT. The other inserts a dummy structure of treatment couch with designated appropriate relative electron density (RED) which is contoured in advance and set as a structure file^20^. The defects are obvious with not only the bias comes from the fusion process, but also that caused by the preset couch features which could deviate greatly from the actual ones. In this work, we measured the attenuation, surface dose, and buildup effect caused by the carbon fiber couch, and then performed radiation dosimetry evaluation with both couch models and compared the accuracy with each other under three different TPS algorithms, pencil-beam (PB), convolution calculation algorithm (CC), and Monte Carlo algorithm (MC), to determinate which model is more superior on calculation accuracy.

## Materials and methods

### uRT-linac 506c medical accelerator

**Figure 1:**
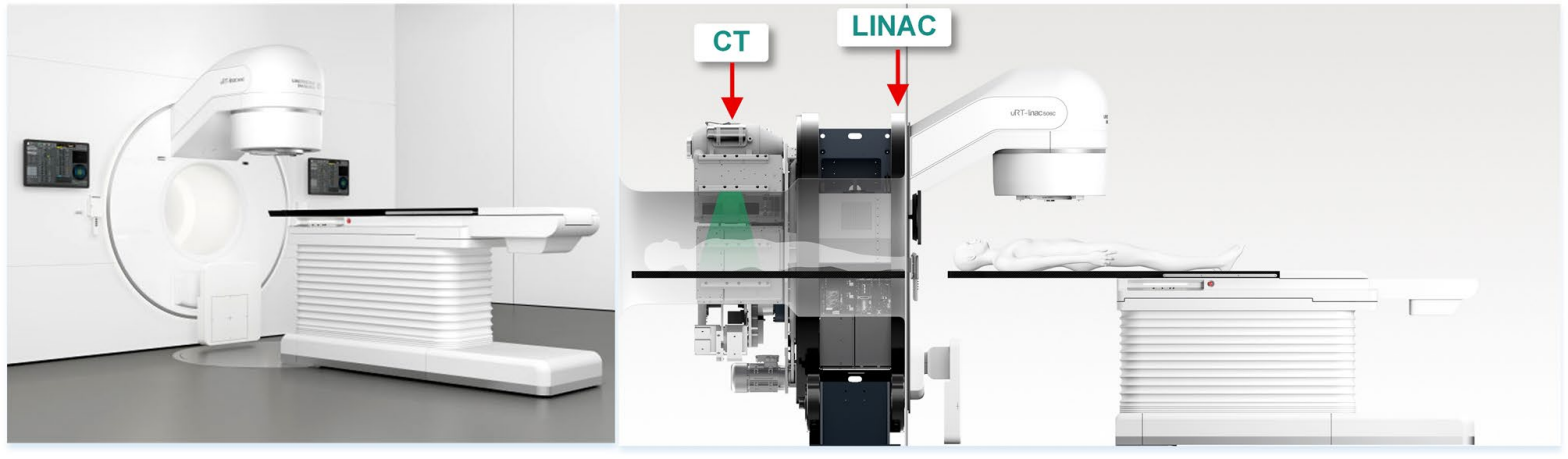
Schematic diagram of uRT-linac 506c linear accelerator (United imaging HealthCare co., LTD., Shanghai) (cited from the commercial brochure with approval).

The uRT-linac 506c (United imaging HealthCare co., LTD., Shanghai) is a novel linear accelerator that combines diagnostic helical CT with a high dose rate intensity modulated accelerator by locating a diagnostic CT behind the gantry sequentially with the same axis (see Fig. 1). This novel structure provides a possibility for high quality image verification, 4D image guided radiotherapy, online adaptive radiotherapy, and so on. Accordingly, the stepping depth of the treatment couch would be much longer than usual, which makes a unique radiation dosimetry effect of it.

### Surface dose and buildup measurements

Surface dose and dose buildup are known closely related to energy, field size and beam geometry^21^. Among various methods, we picked the film method for the simplicity and high spatial resolution, to measure the surface dose and buildup, which was performed with the water equivalent phantom and EBT film. The film was inserted between phantoms with clamps holding position for eliminating air gaps. Before measurement, the film was calibrated with ionization chamber. In order to compare the surface dose with and without the effect of treatment couch, 500MU of 6MV photon was delivered from gantry angle 180° and 0°, respectively. Different field size from 5 × 5 to 25 × 25 cm^2^ were chosen, and the source to surface distance (SSD) was set to 100 cm.

### Couch attenuation measurement

A digital electrometer (UNIDOS E, PTW) and the finger type waterproof ionization chamber (30013, PTW) were used to make charge measurements in the solid water. The standard solid water was used to measure the dose of isocenter point, of which the dimension was 30 cm × 30 cm × 1 cm, and up to 10 cm in thickness by multi-layer stacking. The ionization chamber was placed in the geometric isocenter holder of phantom, which was aligned longitudinally with the treatment couch.

For the absolute point dose measurement, PTW30013 ion chamber was used with sensitive volume of 0.6 cc, the voltage was set to + 400 V for all point measurement, and the temperature and atmospheric pressure were set to 24 °C and 1014.2 mbar, respectively. According to the AAPM TG176 report for the couch attenuation measurement, the radiation field of 10 cm × 10 cm was selected with 6 MV photon. Radiation beams with two opposite directions were performed for measuring the attenuation of couch for various angles, one was set gantry positions at 5°intervals from 180° to 110°, for which radiation passed through the couch, the measured dose value was designated as D_m_; the other was set gantry positions at 5°intervals from 360° to 290°, for which radiation avoids the couch, the measured dose value was designated as D_r_. The couch attenuation factor (*F*) was calculated with the Eq. (1) shown below:

1 $$F = \frac{{D_{r} - D_{m} }}{{D_{r} }} \times 100\%$$

where D_m_ represents the dose measured with the beam passing through the treatment couch and D_r_ represents the dose measured with gantry angle 360°–290° while the beam avoids the treatment couch.

**Figure 2:**
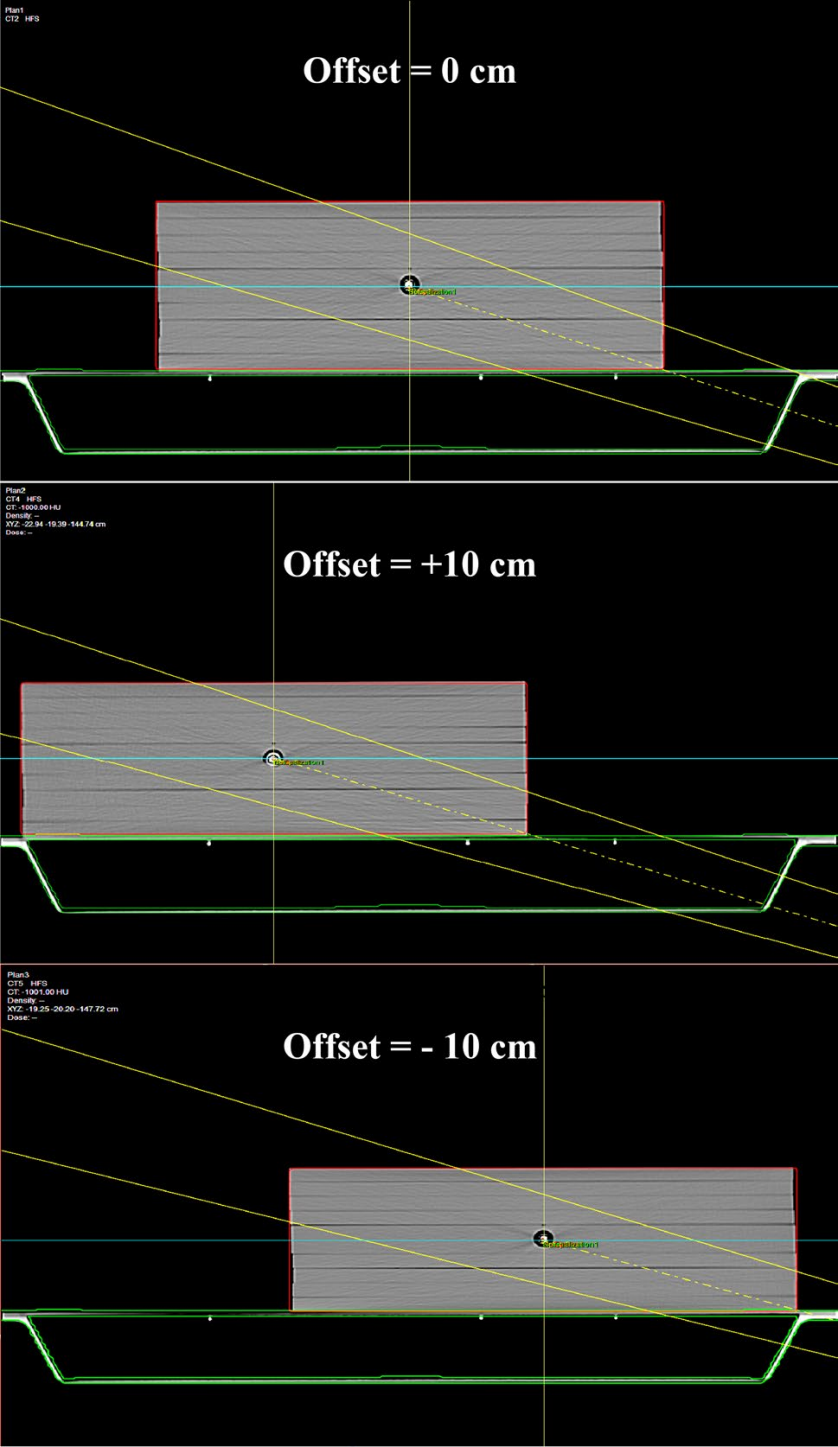
Diagram of solid water phantom at different positions of treatment couch, offset = 0 cm, offset =  + 10 cm, offset =  − 10 cm, respectively.

In order to calculate attenuation at different sites of the carbon fiber couch, the water phantom was located on different positions of the couch, which were in the middle (offset = 0 cm), at the right-side (offset =  + 10 cm), and at the left-side (offset =  − 10 cm), respectively (see Fig. 2).

### Couch modeling in uRT-TPS and dose calculation properties

Providing an diagnostic CT scanning dataset of the carbon fiber couch is a prominent feature of the uRT-linac 506c accelerator, which is crucial for the precise evaluation of the dose distribution caused by treatment couch in uRT-TPS (Ver1.0, United imaging HealthCare co., LTD.). In this work, we first calculated the dose distribution based on the diagnostic CT scanning data of the couch derived from this novel accelerator and set it to be a standard reference as model A due to its authenticity. However, in practice, another method that a dummy structure of treatment couch contoured in advance and calculated in uRT-TPS is widely used, we designated this method as model B. We calculated and measured the dose distribution for both model A and model B with three different algorithms build-in uRT-TPS, called PB, CC, and MC, and compare them to illustrate the deviation between model B and our reference model A.

**Figure 3:**
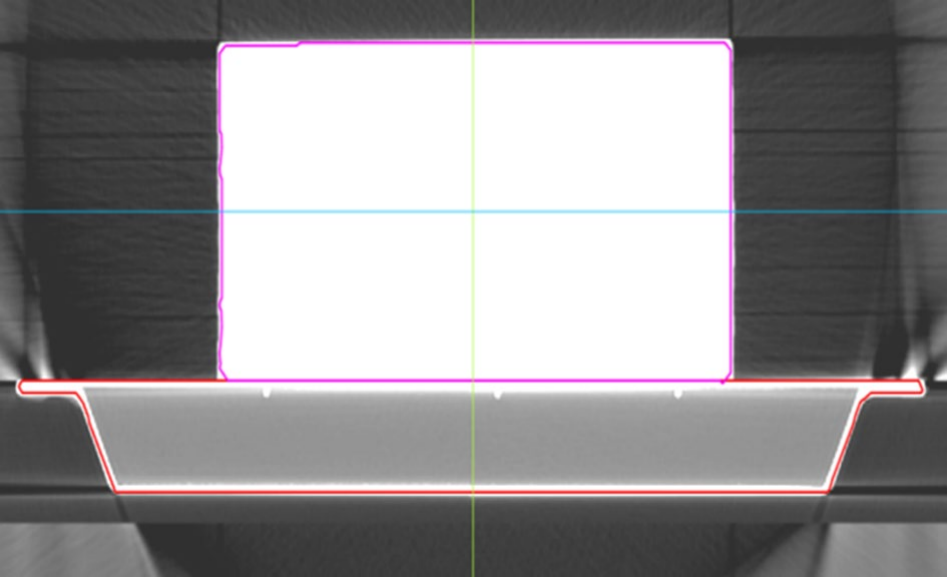
The structures of the carbon fiber couch and phantom dataset that was scanned by the diagnostic CT scanner integrated with the accelerator.

The carbon fiber couch on the uRT-linac 506c accelerator is 225 cm in length, 52.5 cm in width, and 5 cm in thickness. For the model A, the CT scanning of the treatment couch along with water phantom on three different positions as the Fig. 3 shown was acquired by the uRT-linac 506c, and the diagnostic CT image dataset was transferred to the uRT-TPS. The isocenter point dose of the phantom under various gantry angles (same as the materials and methods 2.3) was calculated with the three algorithms. For the model B, a preset dummy structure with designated uniform relative electron density (RED) value 0.26 of treatment couch was introduced and replaced the couch structure in the CT simulation images of the model A, and calculations were performed exactly the same as those of the model A. The dose grid resolution was set to 1.5 mm × 1.5 mm, and the dose deviation (DD) and attenuation factor (*F*_*TPS*_) of the couch in the TPS were calculated with the Eq. (2).

2 $$DD = \frac{{D_{C} - D_{m} }}{{D_{m} }} \times 100\%$$

where *DD* is the dose deviation value between uRT-TPS calculated value and actual measured value; *D*_*c*_ is the uRT-TPS calculated dose value; *D*_*m*_ is the actual measured dose value.

## Results

### Surface dose and buildup measurements

**Figure 4:**
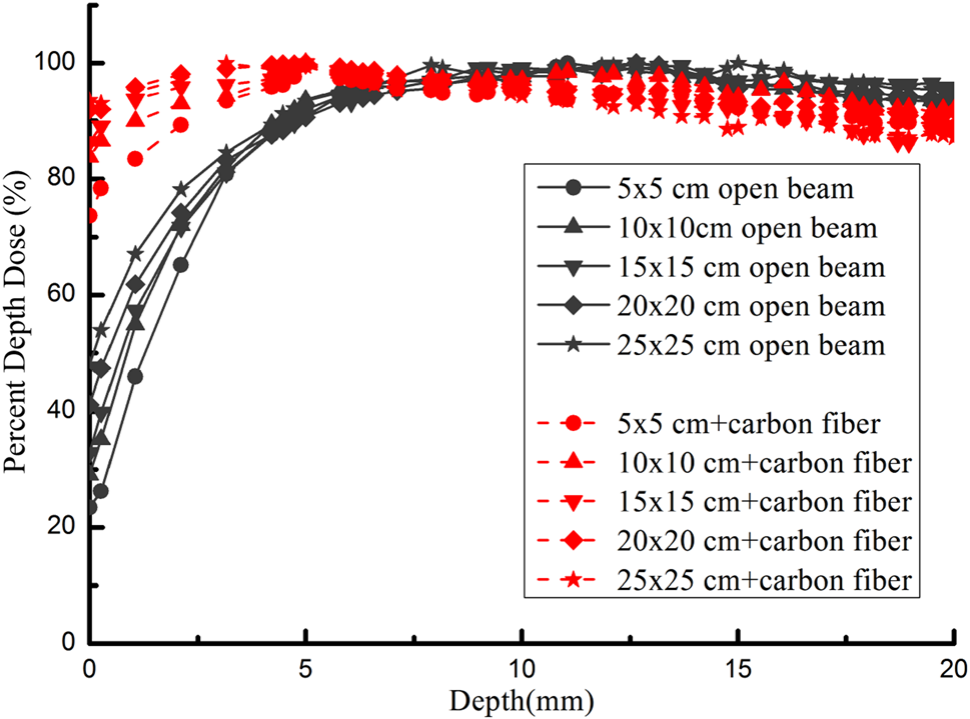
The comparison of the measured percent depth doses (PDDs) curves between open beam irradiation and those with carbon fiber couch involved for different field sizes.

The percentage dose depths (PDDs) for 6 MV photon open beam irradiation and those involved with carbon fiber couch under different fields size ranged from 5 × 5 to 25 × 25 cm^2^ were measured and demonstrated in Fig. 4. As the results shown, the surface dose in buildup region increased with the field size for open beam and those with carbon fiber couch. For open beam irradiation, the surface dose ranged from 23.40 to 41.15% with the field size from 5 × 5 to 25 × 25 cm^2^, while ranged from 73.74 to 93.56% for those with carbon fiber couch. As we can easily know from the data, compared with the open beam, the surface dose in buildup region remarkably increased when carbon fiber couch involved, and the depth of maximum dose varied towards the surface with field size increased. Table 1 showed that the surface dose was increased at least 97.94% for 25 × 25 cm^2^ field and 188.83% for 10 × 10 cm^2^ field with the carbon fiber couch involved compared with those without.

**Table 1:** Relative surface dose in various field sizes for 6 MV photon with and without carbon fiber couch (CFC).

Field size (cm^2^)	Surface dose (%)		Increased percentage of the relative surface dose (%)
	Without CFC	With CFC	
5 × 5	23.54 ± 1.00	73.74 ± 1.15	213.25
10 × 10	29.02 ± 0.45	83.82 ± 1.02	188.83
15 × 15	32.06 ± 0.33	86.61 ± 0.82	170.15
20 × 20	41.27 ± 0.49	91.96 ± 1.04	122.83
25 × 25	47.29 ± 0.38	93.51 ± 0.97	97.94

### Couch attenuation measurements

**Figure 5:**
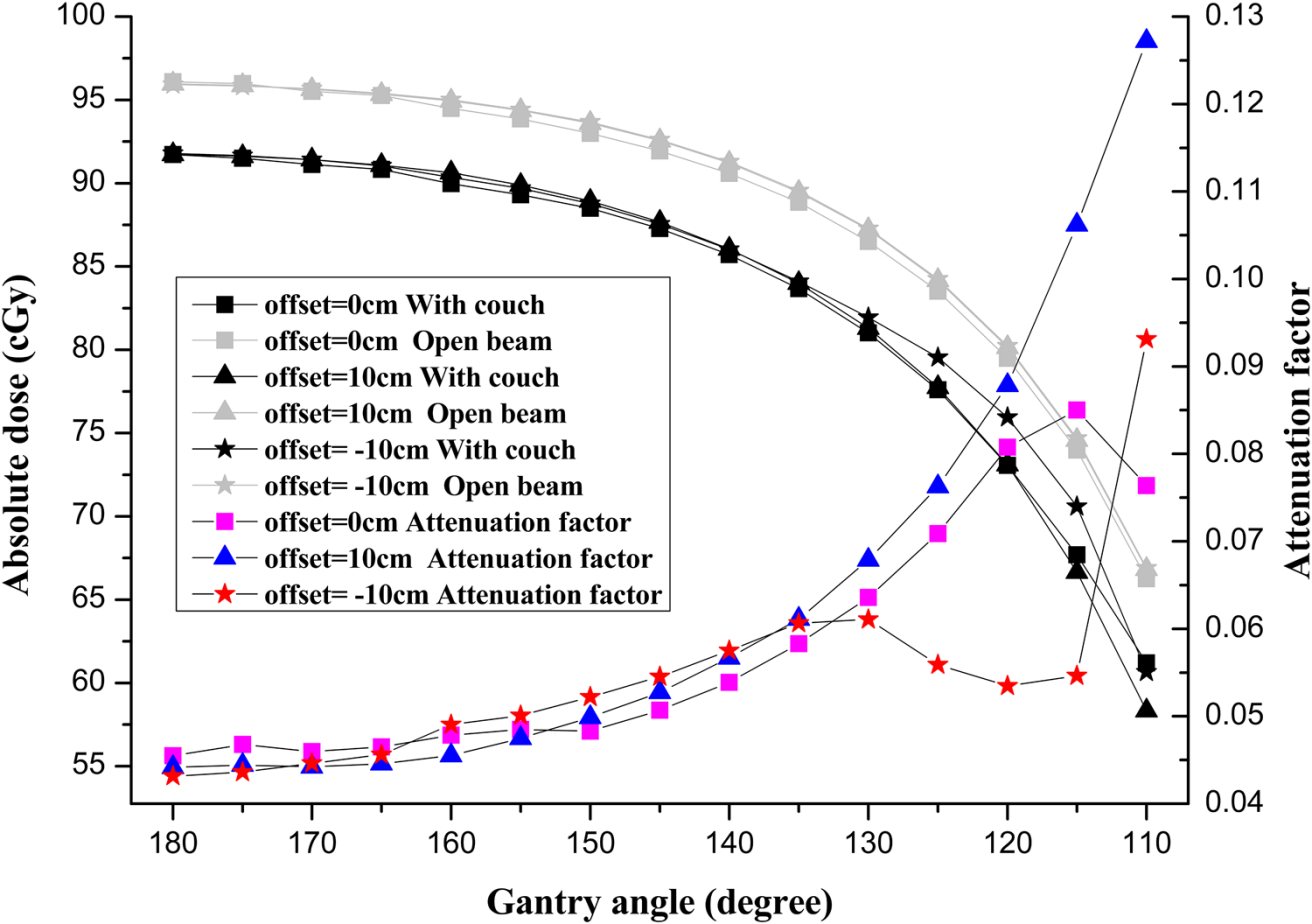
The attenuation profiles with the treatment couch at different positions.

The variation in attenuation of carbon fiber couch for different positions as a function of gantry angle were demonstrated in Fig. 5. At different phantom positions (offset = 0, + 10, − 10 cm), the attenuation factors for 6 MV photon beam at gantry angle 180° were 0.044, 0.044, and 0.043, respectively. With the gantry angles ranged from 180° to 130°, the attenuation factors for the three positions were ascending with the decrease of the angle, and there was no much difference for this sector among three positions. Within the sector ranged from 130° to 110°, the variation of attenuation curves for three positions appeared to be remarkably different. For the curve of offset = 0, the attenuation factor increased with the reduction of gantry angle at first, but turned sharply downwards at the gantry angle 115°, and the max attenuation value for offset = 0 was reached up to 0.085. For the curve of offset =  + 10 cm, the attenuation factor showed continuous upward trend all the way to 0.127. For the curve of offset =  − 10 cm, the attenuation factor displayed a mild ascending with the reduction of gantry angle at first, then gently descended from angle 130° to 115°, and then turned sharply ascending at angle 115°, reaching the maximum value up to 0.093 at the angle 110°.

### The dose deviation between measurements and uRT-TPS calculations

**Figure 6:**
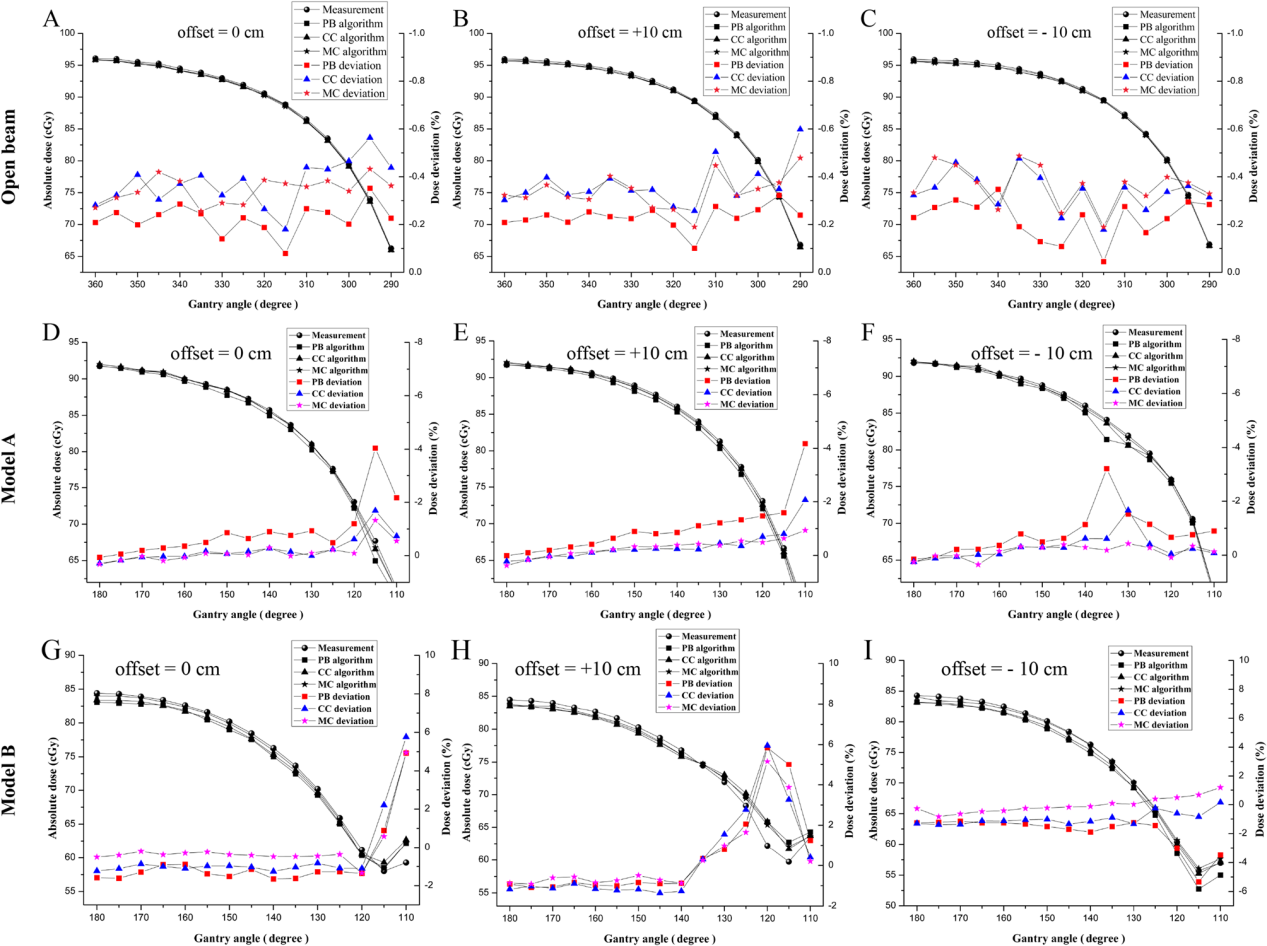
The diagrams illustrating the dose deviations between TPS calculation with three different algorithms and actual measured isocenter dose at different positions for model (**A**) and model (**B**). (**A**)–(**C**) The dose deviation for the open beam irradiation in model A with different positions, offset = 0 (**A**), offset =  + 10 cm (**B**), and offset =  − 10 cm (**C**); (**D**)–(**F**) The dose deviation for irradiation with couch passing through in model (**A**) with different positions, offset = 0 (**D**), offset =  + 10 cm (**E**), and offset =  − 10 cm (**F**); (**G**)–(**I**) The dose deviation for irradiation with dummy couch structure inserted in model (**B**) with different positions, offset = 0 (**G**), offset =  + 10 cm (**H**), and offset =  − 10 cm (**I**).

The results of dose deviation between measurements and uRT-TPS calculations were shown in Fig. 6A–C for open beam irradiation in model A, in Fig. 6D–F for those with couch involved in model A, and in Fig. 6G–I for those with dummy couch structure involved in model B utilizing the three algorithms, PB, CC, and MC, respectively.

In Fig. 6A–C for the open beam irradiation in model A with different positions, offset = 0 (Fig. 6A), offset = + 10 cm (Fig. 6B), and offset = − 10 cm (Fig. 6C), without involving of the carbon fiber couch, the actual measured isocenter dose in water phantom and the uRT-TPS calculated counterparts were demonstrated and the curves went pretty close, which meant uRT-TPS calculations reflected the actual dose well if absent of the couch effect, no matter which algorithm used. The deviations calculated according to the equation (2) for various gantry angles ranged from − 0.08 to − 0.56% for position offset = 0, from − 0.10 to − 0.60% for position offset= +10 cm, from − 0.04 to − 0.49% for position offset = − 10 cm, indicating pretty high consistency. There was no much difference among three algorithms, the average deviation was overall less than 0.37%.

In Fig. 6D–F for irradiation with couch passing through in model A with different positions, offset = 0 (Fig. 6D), offset = + 10 cm (Fig. 6E), and offset = − 10 cm (Fig. 6F), the actual measured isocenter dose in water phantom and the TPS calculated counterparts were demonstrated with the three algorithms, and the curves went separately with considerable gaps indicating couch involvement did make bias for TPS calculation. The deviations for various gantry angles ranged from + 0.32 to − 4.04% for position offset = 0, from + 0.01 to − 4.18% for position offset = + 10 cm, from + 0.02 to − 3.20% for position offset = − 10 cm, revealing deviation increased significantly compared with those with open beam irradiation, especially for angle section from 120° to 110°. From angle 180° to 120°, the deviation increased gradually with the decrease of the angle, except for angle 135° on the position offset = − 10 cm, PB algorithm showed sharply increased deviation (Fig. 6F). In general, the dose calculated by PB made the largest deviation (average deviations were 0.50–0.82%), and the deviation derived from CC (average deviations were 0.13–0.39%) and MC (average deviations were 0.13–0.28%) were very similar for the angle sector 180°–120°, and MC still kept low deviation even for the angle sector 120°–110°, which indicated MC were the best one with high accuracy among the three algorithms.

In Fig. 6G–I for irradiation with dummy couch structure inserted in model B with different positions, offset = 0 (Fig. 6G), offset = + 10 cm (Fig. 6H), and offset = − 10 cm (Fig. 6I), the actual measured isocenter dose in water phantom and the uRT-TPS calculated counterparts were demonstrated with the three algorithms. The deviations for various gantry angles ranged from − 0.20 to + 5.76% for position offset = 0, from + 0.21 to + 5.95% for position offset = + 10 cm, from + 0.02 to − 5.34% for position offset = − 10 cm. From angle 180° to 120°, the deviation was steady with the decrease of the angle, except for the position offset = + 10 cm (Fig. 6H), where it started to increase sharply from the angle 140° up to the + 5.95% at the angle 120°, then dropped dramatically at the angle 110°. From angle 180°–120°, the average deviations of PB (0.97–1.35%) and CC (1.04–1.25%) were similar, while that of MC (0.38–0.79%) were remarkably smaller. From angle 120°–110°, the deviation varied with dramatic wide range. Among the three algorithms, MC still was the best one to keep relative low deviation even for the angle section 120°–110°.

**Figure 7:**
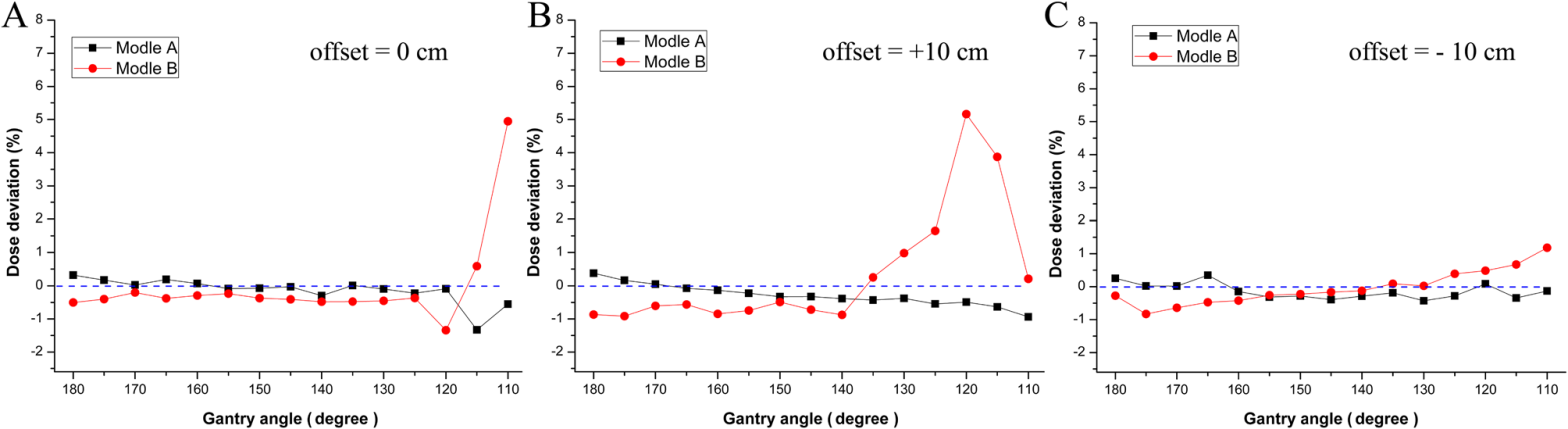
The comparison of the deviation between TPS calculation (MC algorithm) and actual measured isocenter dose for model (**A**) (with authentic CT scanning data of the couch) and model (**B**) (with inserted dummy couch structure data). The blue dash lines indicate actual measured isocenter dose (deviation = 0).

Since the MC was the most accurate algorithm in our study, we compared the deviation between TPS calculation with only MC algorithm and actual measured dose for model A (with diagnostic CT scanning data of the couch) and model B (with inserted dummy couch structure data), with the aim to determinate which couch model was more accurate to make less deviation in uRT-TPS calculation. Figure 7 showed that model A made less and steadier deviation compared with model B, which suggested that the diagnostic CT scanning data of the treatment couch made less deviation in uRT-TPS.

## Discussion

Accurate radiation dose delivery is crucial for the successful execution of modern precise radiation therapy. The radiotherapy outcomes demands accuracy to generally within 3–5% based on the goal of maximizing tumor control and minimizing injury to adjacent OARs^22, 23^. ICRU24 reported deviation of delivered dose more than 5% could lead to failure of local control or unexpected complications. In AAPM TG176^8^, it’s summarized that various external devices involved in radiotherapy make significant deviation for the dose attenuation and distribution, among them, the treatment couch contributes the most.

The diagnostic helical CT scanner, which is located on the same axis with the linear accelerator, shares the same carbon fiber couch with the accelerator. This novel structure provides a possibility to make the scanned couch dataset be the same one between the simulation and dose delivery. Therefore, the evaluation of the detailed effect of the couch on dose attenuation and distribution would be helpful for those who might use this accelerator in the future. With this purpose, this research evaluated the effect of the couch on the surface dose and buildup effect, and attenuations from various gantry angles. We also revealed the superiority of the diagnostic CT scanning dataset of the couch over a dummy structure inserted to be more accurate on TPS calculation.

The surface dose and buildup effect are the major concern for the employment of a treatment couch. If those effects are ignored, it may cause unexpected serious skin injury and target dose reduction. In this study, the dose deviation bringing in by the treatment couch of the uRT-linac 506c was generally consistent with previous research. For example, Seppälä et al.^4^ measured that the increased percentage of relative skin dose with involvement of eight different CFCs varied from 73.86 to 180.11% under the context of field size 10 cm × 10 cm for 6 MV photon, the best ones were Varian grid insert and Qfix kVue DoseMax which were both grid-based, those of most solid CFCs were similar to our data. For some counterparts, the surface dose effect in our work showed apparent superiority, for instance, David et al.^24^ measured that the increased skin dose with involvement of the iBEAM evo CFC was 513%, and Sheykhoo et al.^25^ reported their data of 514.53% increased skin dose with the 550 TXT CFC under the same context of 10 cm × 10 cm for 6 MV photon, while that of uRT- linac 506c was 213.25%. The effect depends on multiple factors including couch design and structure, material, photon beam quality, and field size as well. The uRT- linac 506c couch is of 5.0 cm in thickness for the whole and 0.5 cm in thickness for the top layer, 1.27 g/cm^3^ in mass density for carbon fiber layer each and 0.07 g/cm^3^ for the inner layer. We assumed that the surface dose was close related with the thickness and mass density of the top layer if regardless of the couch structure. Meydanci et al.^16, 17^ reported similar increased relative skin dose (148%) to ours for the RM2/4 CFC of which the thickness (1.8 g/cm^3^) and mass density of the top layer (0.6 cm) resembled those in our study.

The attenuation factor of 6 MV photon beam by the CFC in this study increased as the gantry angle of incidence decreased from 180° to 110°. The results were similar to those of other institutes^6, 17, 26^. We can see in Fig. 5 that the attenuation factor showed smooth and mild ascending as the angle decreased from 180° to 130° for all three positions with no much difference, however, from 130° to 110°, it’s demonstrated distinct variation for different positions, offset = 0 cm and offset =  + 10 cm showed sharp ascending, but offset =  − 10 cm showed dramatic fluctuation, which is considered to be due to several reasons. First, the rectangular solid water phantom was used in this study, the path length of beam through the phantom was different as the angel of incidence varied, which could make dramatic change at specific angle. Second, the keel which was made of carbon fiber and used for strengthening of the rigidity of CFC could also contribute attenuation for specific position. Third, the geometrical structure of CFC especially the edge made dramatic fluctuation of the attenuation curve, which was unique for specific CFC, when the beam went through the edge. Regardless of all these factors, the attenuation of perpendicular beam at angle 180° in this study was 4.4%, while it’s 4.7% for Varian’s Exact Couch Top (ECT, Varian Medical Systems)^27^, 3.0% for RM2/4 CFC (Reuther Medizin Technik)^17^, and 2.7% for iBEAM evo CFC (Medical Intelligence)^24^, which was thought to be related to the thickness and mass density of the carbon fiber layer.

The couch scanned in the simulation CT data set was usually not the exact one with which patients were treated, and of course not the one on which treatment planning and calculation was performed as well, the deviation between them was commonly major. Therefore, in practice, two methods were employed to deal with it, one is that the couch of simulation CT was replaced with a separate CT scan of the exact couch on the treatment accelerator by fusion, obviously, the bias occurred for these two different couches would fail to match perfectly; the other is that a dummy structure of the treatment couch was delineated with designated RED to replace the couch in simulation CT scan. The defects were also the unmatched fusion, and further the homogeneous RED artificially preset could not reflect the actual situation making bias for TPS calculation. The second one was practically more commonly used. The uRT-linac 506c with an integrated diagnostic CT scanner could conquer the problems. The diagnostic CT scanning dataset can be used directly for target contouring and subjected to treatment planning, which also facilitate the online adaptive radiotherapy. By this means, the authentic CT scanning dataset of the treatment couch with real RED could be used directly for TPS calculation, without introducing bias by fusion or dummy structure with artificial RED. In this study, we termed this model as model A, compared with the dummy structure defined as model B. At present, the uRT-linac 506c is the only accelerator capable to acquire the diagnostic CT scanning dataset of the treatment couch with real RED, which is a promising platform for online adaptive radiotherapy.

In this study, MC appeared to be the most accurate one among all the three algorithms, which constantly made the least deviation from the actual measured dose, while the PB algorithm generally presented the largest deviation with the worst accuracy in our TPS calculation. This result was consistent with Krieger T. et al.’s study^28^. The PB algorithm is suitable for homogeneous phantom with high accuracy, but the accuracy worsens badly for heterogeneous phantom. It is all due to that it only takes into account the thickness of phantom, without consideration of the scattering effect around the calculation point and the imbalance of lateral electrons on the interface between heterogeneous parts^29–31^. CC algorithm possesses higher accuracy for heterogeneous phantom than PB, for it involves not only thickness, but also the scattering effect of material interface, and lateral electronic imbalance as well, but it still lose some accuracy if encounters interface with too much difference^28, 29, 31^. The principle of MC algorithm is to simulate impact interactions among large numbers of particles and phantom materials or human tissues, calculating and recording the released energy of each interaction of mass quantity and further accumulating the site-specific deposited energy to acquire a complete dose distribution^28, 30, 31^. So theoretically, the MC algorithm should be the one with the highest accuracy even for heterogeneous phantom or tissue, and was employed widely as gold standard for TPS calculation^28^, however the vast amounts of calculation was not feasible for practical TPS performance, so it’s commonly utilized as a simplified version varied among vendors and systems.

To evaluate the difference of calculation accuracy between the couch dataset from the authentic CT scanning and that from the dummy structure used in uRT-TPS, the MC calculated dose at isocenter was compared with the actual measured dose (shown in Fig. 7). The authentic CT scanning dataset appeared to be steadier and of less deviation with satisfying accuracy, which indicated that the diagnostic CT scanning dataset would be a better choice. This result suggests that the diagnostic CT scanner equipped with an accelerator could be the trend of future development.

## Conclusion

The uRT-linac 506c is an innovative accelerator, which is equipped with a diagnostic helical CT scanner that locates directly behind the accelerator. In this study, we measured the attenuation, surface dose, and buildup effects of the carbon fiber couch equipped on, which were consistent with most counterparts. So far, as an innovative structure, uRT-linac 506c is currently the only accelerator capable to acquire the authentic CT scanning dataset of the treatment couch with real RED. This authentic CT scanning dataset was proved to have achieved higher accuracy than the model with a dummy couch inserted. This results suggest that integrating a diagnostic CT scanner and an accelerator on the same axis could help reduce the dose deviation about the carbon fiber couch, which is a promising platform for online adaptive radiotherapy in the future.

## Data Availability

Please contact the corresponding author for data requests.

## Abbreviations

EBRT: External beam radiotherapy
IMRT: Intensity modulated radiotherapy
IGRT: Imaged guided radiotherapy
VMAT: Volumetric arc therapy
PTV: Planning tumor volume
RED: Relative electron density
PB: Pencil-beam algorithm
CC: Convolution calculation algorithm
MC: Monte Carlo algorithm
SSD: Source to surface distance
DD: Dose deviation
PDD: Percentage dose depth
TPS: Treatment planning system
OAR: Organ at risk
CFC: Carbon fiber couch
